# Pharmacological basis for the traditional use of *Myrica salicifolia* root extract in gut motility disorders via cholinergic and calcium/PDE modulation

**DOI:** 10.1016/j.jtcme.2026.05.001

**Published:** 2026-05-28

**Authors:** Najeeb Ur Rehman, Mohd Nazam Ansari, Aman Karim, Abdullah Rabea Tashah, Hussain Mohammad Daak, Khalil Y. Abujheisha, Muneeb Ur Rehman, Wasim Ahmad

**Affiliations:** a Department of Pharmacology and Toxicology, College of Pharmacy, Prince Sattam Bin Abdulaziz University, Al-Kharj, 11942, Kingdom of Saudi Arabia; b Department of Biotechnology, National University of Medical Sciences (NUMS), Rawalpindi, Pakistan; c Department of Basic Medical Science, Faculty of Medicine and Health Science, Palestine Polytechnic University, Hebron, Palestine; d Key Laboratory for Green Chemical Process of Ministry of Education Hubei Key Laboratory of Novel Reactor and Green Chemical Technology, Hubei Engineering Research Center for Advanced Fine Chemicals, School of Chemical Engineering and Pharmacy Wuhan Institute of Technology, 206 1st Rd Optics Valley, East Lake New Technology Development District, Wuhan, Hubei, 430205, China; e Department of Pharmacy, Mohammed Al-Mana College for Medical Sciences, Dammam, 34222, Saudi Arabia

**Keywords:** *Myrica salicifolia*, *Muscarinic*, Antispasmodic, Ca^++^ channel blocker, PDE inhibitor

## Abstract

**Background and aim:**

This study was carried out to evaluate the possible gut modulatory and antibacterial activities of *Myrica salicifolia* root extract (Ms.Cr).

**Experimental procedure:**

Gas Chromatography-Mass Spectrometry (GC-MS) analysis was conducted for chemical profiling, while FTIR analyzed the chemical bonds. Mice were used for *in vivo* experiments, while possible pharmacodynamics were explored in rabbit jejunal tissues.

**Results and conclusion:**

GC-MS analysis identified 16 metabolites, while mice administered with lower doses of Ms.Cr (50 and 100 mg/kg) causes increase in the count of wet feces and the total number of fecal outputs, which was significantly reduced in the pre-atropinized mice. At higher doses of 200 and 400 mg/kg, Ms.Cr showed respective protection of 40% and 80% in mice in castor oil induced diarrhea. In jejunal tissues, lower concentrations of Ms.Cr increased the spontaneous contractions followed by complete relaxation at higher concentrations. Further, Ms.Cr caused concentration-dependent (1.0-5.0 mg/mL) relaxation of high K^+^-induced contractions, similar to papaverine. Preincubated jejunal tissues with Ms.Cr shifted the Ca^++^ concentration-response curves (CRCs) towards right with suppression of the maximum response, similar to verapamil, thus confirming Ca^++^ channel blocking activity. Similar to papaverine, preincubation of tissues with 3 and 5 mg/mL of Ms.Cr, caused shift in isoprenaline CRCs towards left, thus showing phosphodiesterase inhibition. Ms.Cr showed an antimicrobial effect against pathogenic gut bacteria including *E. coli*, *Sh. sonnei*, *S. typhimurium* and ESβL (extended-spectrum beta-lactamase)-producing *E. coli*. Thus, this research provides a solid foundation for the therapeutic uses of *M. salicifolia* in gut motility-related disorders.

## Highlights

• Extract of *Myrica salicifolia* (Ms.Cr) produced gut modulatory effects in rodents.
• In the GC-MS analysis, 16 metabolites were identified in Ms.Cr.
• Lower doses of Ms.Cr produced gut stimulant effect in *in vivo* and *ex vivo*.
• Higher doses of Ms.Cr produced anti-diarrheal and antispasmodic effect in rodents.
• Gut modulatory effect is caused by cholinergic, phosphodiesterase and Ca^++^ channels.

## Graphical abstract

**Figure:**
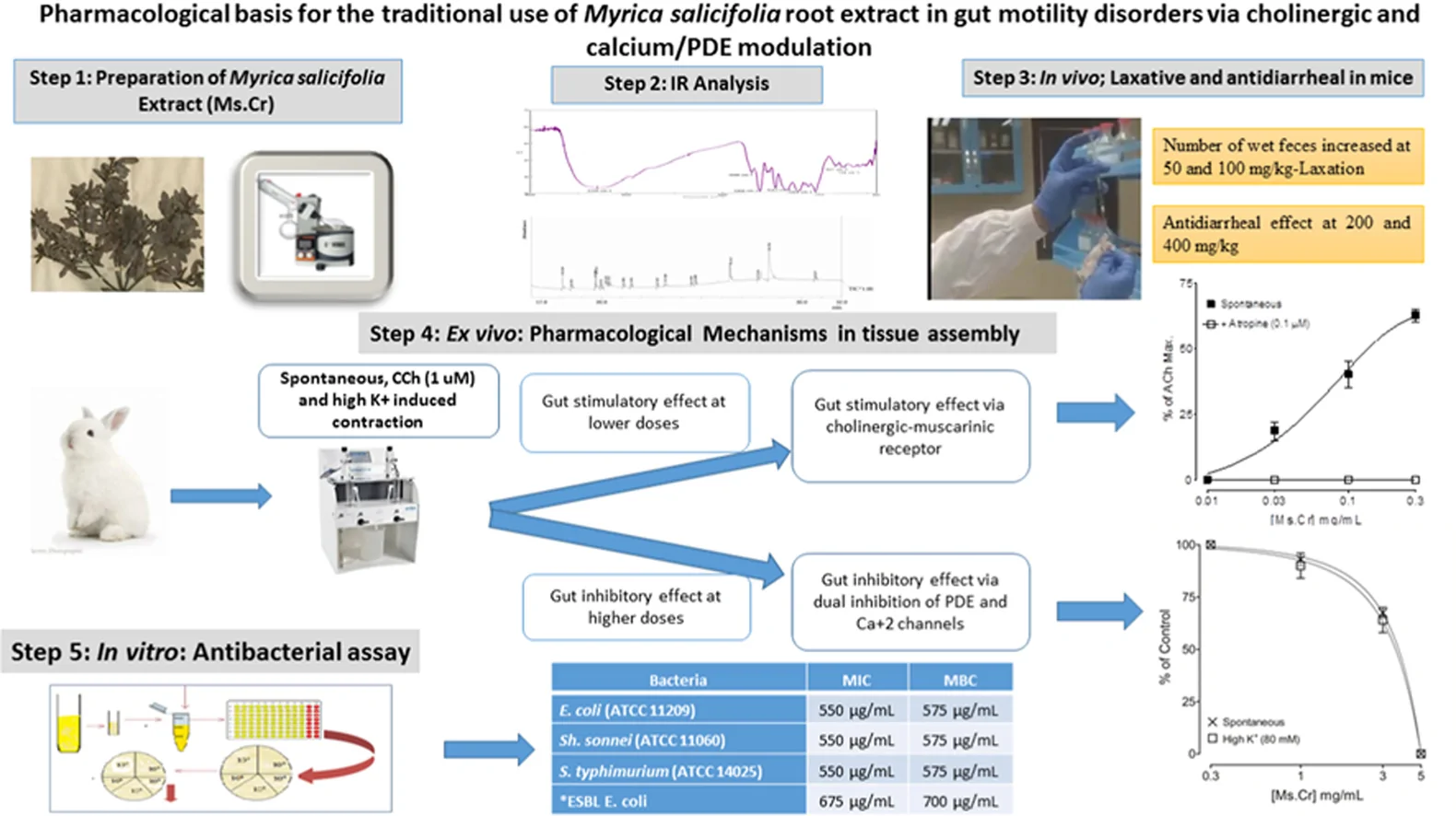

**Table:** List of abbreviations

μ	Micro
μM	Micromolar
ACh	Acetylcholine
ATCC	American Type Culture Collection
Ca^++^	Calcium ion
CaCl_2_	Calcium chloride
CCB	Calcium channel blocker
CCh	Carbachol
CO_2_	Carbon dioxide
CRCs	Concentration-response curves
EC_50_	Median effective concentration
EDTA	Ethylenediamine tetracetate
ESβL	Extended Spectrum Beta Lactamase
FTIR	Fourier Transform Infra-Red Spectroscopy
GC-MS	Gas Chromatography-Mass Spectrometry
K^+^	Potassium ion
MIC	Minimum Inhibitory Concentration
mL	Milliliter
mM	Millimolar
Ms.Cr	*Myrica salicifolia* crude extract
n	Number of observations
NIST	National Institute of Standards and Technology
O_2_	Oxygen
^o^C	Centigrade
PDE	Phosphodiesterase enzyme
SEM	Standard error of mean
WHO	World Health Organization

## 1 Introduction

Diarrhea and constipation are two opposite disease states of the gut that affect major populations worldwide. Currently, the available drugs to treat constipation often cause diarrhea, and anti-diarrheal agents are often associated with constipation as a side effects if consumed at higher doses.^1^ According to WHO report, three-quarters of the world population depends on natural products due to their safety, cost-effectiveness, and the presence of synergistic and/or side effects neutralizing components.^2^ Therefore, it is necessary to explore alternative therapies derived of natural origins.

Infections of the gastrointestinal tract by various forms of gut pathogens including bacteria, viruses, parasites, and fungi cause inflammation and thus results in diarrhea or gastroenteritis. The current approach to combat multidrug-resistant (MDR) bacteria involves the use of a combination of synthetic antibiotics and natural antibacterial agents.^3^ *Myrica* is one of the largest genera in the family of Myricaceae, comprising about 760 species.^4^ The genus *Myrica* is native to warm temperate regions, as well as southern and tropical West Africa, East and Southeast Asia, the Macaronesia Islands, and subtropical South, Central, and North America.^5^ Different species of *Myrica* have been used in indigenous communities in Brazil for the treatment of digestive problems, bronchitis, asthma, hypertension, and diabetes mellitus.^4^ *M. salicifolia* is a shrub usually 1 m in height but can grow up to 20 m tall aromatic and resinous tree.^6^^,^^7^ It is usually found in humid lower highlands between 1800 and 3000 m in altitude and distributed in Saudi Arabia, Southeast Africa, and Ethiopia.^7^, ^8^, ^9^ It is commonly known as Shinet (Amharic) in Ethiopia.^10^

Root infusion of *M. salicifolia* has been used for the treatment of gastrointestinal disorders, bilharzia, and malaria,^9^^,^^11^ while additional studies show the therapeutic benefits of its roots and bark in diarrhea, pneumonia, chest congestion, nervous disorders, pain, headache, inflammation, and hypertension.^9^^,^^12^ The chloroform and water extracts of *M. salicifolia* have been reported for antibacterial effects against selected strains such as *K. pneumonaie*, *E. coli*, *S. typhi,* and *P. aeruginosa*.^7^ Njung et al. reported strong analgesic activity of its root extract in mice.^6^ Despite the ethnobotanical claims of the root extract of *M. salicifolia* in the treatment of digestive diseases, there is no detailed scientific data to support its effectiveness in addressing dual disease states of the gut i.e. constipation and diarrhea. Therefore, this study aimed to provide a scientific base for the gut modulatory effects of the root extract of *M. salicifolia* in rodents.

## 2 Materials and methods

### 2.1 Ethics statement

The study was approved by the Standing Committee of Bioethics Research at Prince Sattam Bin Abdulaziz University (Reference # SCBR-394/2024). Ethical principles were followed in the *in vivo*, *ex vivo* and *in vitro* experiments.^13^

### 2.2 Chemicals

All chemicals used were of analytical grade and purchased from sources specified; acetylcholine perchlorate (ACh), carbachol (CCh), verapamil, loperamide, isoprenaline, papaverine, and atropine were acquired from Sigma Company, St. Louis, MO, USA. Physiological buffer (Tyrode) for isolated tissue experiments was prepared using the following salts: KCl (Sigma Co.), CaCl_2_, glucose, MgCl_2_, sodium bicarbonate, potassium dihydrogen phosphate, and NaCl (Merck, Germany). Castor oil used as oral gavage in the *in vivo* experiments was purchased from a local pharmacy in Al-Kharj.

### 2.3 Plant material and extraction

The roots of *M. salicifolia* were collected on a single day from the Genfoquch area in the northern part of Gondar town, Amhara Region, Ethiopia, in December 2018 to minimize seasonal and geographical variations affecting plant metabolite composition. The plant was authenticated by Abiyu Enyew (botanist), Department of Biology, College of Natural Sciences, University of Gondar, and a specimen (voucher no. YK001) has been deposited at the Herbarium of the Department of Biology, College of Natural Sciences, University of Gondar. The powdered roots of *M. salicifolia* (700 g) were soaked in 80% methanol (v/v) for 72 h, with occasional shaking, and the process was repeated thrice. All the three filtrates were combined and filtered using Whatman No. 1 filter paper. The resulting hydroalcoholic filtrate was dried in an oven at a temperature not exceeding 40 °C and was stored in a refrigerator for future use. The FTIR and GC-MS analysis of the extract was conducted to obtain to fingerprint profiles of the extract.

### 2.4 GC-MS analysis

In the chemical constituents analysis of Ms.Cr using GC-MS, the separation of phytoconstituents was carried out using an Agilent GC-MS system (Agilent Technologies, Santa Clara, CA, USA) equipped with an HP 5MS column (30 m × 0.25 mm × 0.25 μm). The extract's constituents were separated under the following conditions: the oven temperature was initially set to 60 °C for 2 min, and then increased by 10 °C per min until reaching 300 °C, which was maintained for 19 min, resulting in a total run time of 32 min. Helium served as the carrier gas at a flow rate of 1.21 mL/min, and the injector temperature was set at 270 °C. The separated constituents were converted into mass peak spectra and identified by comparing them with the mass spectra in the built-in National Institute of Standards and Technology (NIST) GC-MS library.^14^

### 2.5 Fourier transform infra-red spectroscopy (FTIR)

The functional group and chemical bonds present in the hydro-alcoholic root extract of *M. salicifolia* were

Analyzed by a Fourier transform infrared (Jasco FT-IR Spectrophotometer, Japan). Briefly, the dried powder of the extract was grounded with crystalline potassium bromide (1:10), and a transparent film was produced by applying pressure. The FTIR spectra were recorded (range 400-4000/cm), and peaks were identified using spectra manager software.^15^

### 2.6 Animals

Adult healthy rabbits (1.0-1.4 kg) were procured from the local Al-Kharj market called, Souq, whereas the Swiss albino strain of mice (25-30 g) available in the Animal Care Unit of the College of Pharmacy, PSAU were used in the *in vivo* study. The temperature in the animal housing facility was maintained at an optimal level of 24 ± 1 °C, with a relative humidity of 55 ± 5%, and an equal interval of light/dark cycle. The animals were provided with water and standard diet pellets ad libitum.

### 2.7 *In vivo* experiments

#### 2.7.1 Laxative study

Following the previous method,^16^ total 42 mice, equally divided into seven groups (n = 6) and were placed individually in cages lined with clean filter paper. After fasting for 6 h, saline (10 mL/kg) was administered by oral gavage (per os [PO]) to the 1st group, labeled as a negative control, while mice of the 2nd group received carbachol (1 mg/kg), a standard drug (Intraperitoneally [IP]). Animals of the 3rd and 4th groups received increasing doses of Ms.Cr (50 and 100 mg/kg, PO), respectively. To determine the mechanism of the laxative effect, animals of the 5th, 6th and 7th groups were pre-treated with atropine (10 mg/kg, IP) an hour before the oral administration of Ms.Cr and/or carbachol. After 18 h, the feces production in all animals were recorded by counting the total number of feces and total number of wet feces per group. The percentage increase in wet fecal spots compared to total fecal number was recorded and statistically compared with saline-treated mice to find laxative effects.^17^

#### 2.7.2 Antidiarrheal study

Twenty mice fasted for 24 h were randomly divided into four groups (n = 5). Mice of the 1st group (negative control) were orally administered with normal saline (0.9% w/v, 10 mL/kg). Following pilot screening for dose selection, the 2nd and 3rd groups (test groups) were treated with two increasing doses (200 and 400 mg/kg, PO) of Ms.Cr, respectively. Animals of group four (positive control) received loperamide (10 mg/kg, PO). Following administration of the relevant drugs, each animal was kept in a separate cage lined with a clean blotting paper to record the diarrheal spots. After 1 h of the administration, each animal was orally administered with castor oil (10 mL/kg) and 4 h of the castor oil dosing, blotting sheets of all the cages were inspected for diarrheal spots by a blind observer. The absence of diarrheal spot was marked as percent protection.^18^

### 2.8 *Ex vivo* experiments

#### 2.8.1 Isolated rabbit jejunum

Overnight fasted rabbits were exposed to cervical dislocation by a blow on their neck in a laying down position, and the euthanasia was confirmed by the absence of ear and corneal reflexes. Their abdomen was cut opened to isolate the central part of the small intestine called, jejunum.^19^ Different segments of the jejunum were cut into 2-3 cm lengths and cleaned off gently from the attached fatty mesenteries. Each isolated tissues was immediately transferred to the organ bath, filled with physiological buffer and the tissue contractile/relaxant responses were recorded by the attached transducer and IOX software installed with the assembly (emkaBath, France). Each tissue baths (20 mL), filled with freshly prepared Tyrode solution (pH 7.4) and aerated with bubbles of carbogen gas while the bath temperature was kept constant at 37 °C by the attached heater. Each tissue was allowed to stabilize for 30 min without adding any drug while the bath medium was replaced with fresh solution every 10 min. After stabilizing, acetylcholine (Ach; 0.3 μM) was added to the bath repeatedly with 5 min interval until a stable band of spontaneous contractions was achieved. Increasing concentrations of Ms.Cr were added into the tissue bath in cumulative fashion to observe any stimulant/relaxant responses at the stable band of spontaneous contractions of jejunum tissue.

#### 2.8.2 Gut stimulatory effects

The lower concentrations of Ms.Cr increased while the higher doses caused decrease in the spontaneous contractions of rabbit jejunum, hence further experiments were planned to know the gut stimulatory mechanism involved. The methods described by Rehman et al. was followed, where the tissues were pre-incubated with atropine (0.1 μM) for 30 min and the stimulant effect of the previously observed doses of Ms.Cr was re-evaluated.^20^ Atropine, being an anti-muscarinic drug,^21^ will block any stimulant effect mediated by activation of muscarinic receptors in the gut.

#### 2.8.3 Gut inhibitory effects

As the higher concentration of Ms.Cr showed gut relaxant effect, further experiments were planned to explore the dynamics of its antispasmodic effect by testing Ms.Cr against high K^+^-induced contractions. Earlier studies show that exposure of the tissues to the higher concentration of KCl (K^+^ > 30 mM final bath concentration) depolarizes the smooth muscles and causes sustained contractions of the tissue by opening L-type Ca^++^ channels.^22^ Hence, any test sample that reverses the high K^+^ (80 mM)-mediated contractions is considered as calcium channel blocker (CCB), whereas an agent that reverses spontaneous and high K^+^-mediated contractions at comparable concentrations is considered as phosphodiesterase enzyme (PDE) inhibitor.^23^^,^^24^

After observing the similar pattern of inhibition of spontaneous and high K^+^-mediated contractions in isolated jejunum by Ms.Cr, additional experiments were conducted to further confirm the CCB and PDE-like activities. For a CCB-like effect, the jejunal tissues were incubated for 45 min in Ca^++^-free Tyrode solution containing EDTA (0.1 mM) to chelate calcium. Further depletion of the tissue intracellular stores of Ca^++^ was made by replacing the Ca^++^-free medium with Ca^++^-free and K^+^-rich Tyrode solution with composition as cited earlier.^23^^,^^25^ After 1 h incubation of the tissue in Ca^++^-free and K^+^-rich medium, increasing concentrations of CaCl_2_ was added exogenously to construct Ca^++^ CRCs in the absence and in the presence of preincubated tissues with increasing concentrations of Ms.Cr. The Ca^++^ CRCs of Ms.Cr was compared with verapamil, which served as a controlled CCB.

To confirm PDE inhibition as additional possible antispasmodic mechanism present in Ms.Cr, inhibitory CRCs of isoproterenol were obtained against CCh-induced contractions in the absence and presence of pre-incubated tissues with Ms.Cr. The leftward shift of isoprenaline inhibitory CRCs towards lower concentrations indirectly indicate the PDE inhibitory-like components present in Ms.Cr, similar to papaverine, a well-known PDE inhibitor.^26^

### 2.9 *In vitro* experiments

#### 2.9.1 Bacterial strains

Antimicrobial assays were conducted using reference strains from the American Type Culture Collection (ATCC) available at the Microbiology Laboratory of the College of Pharmacy, PSAU, Saudi Arabia. The standard bacterial strains used were as follows: *E. coli* (ATCC 11209), *S. typhimurium* (ATCC 14025), and *Shigella sonnei* (ATCC 11060). In addition, extended-spectrum beta-lactamase (ESβL)-producing *E. coli* was tested as a clinical pathogen. ESβL-producing *E. coli* was isolated and identified by Dr. Abujheisha with the cooperation of King Khalid Hospital (Study IRB- 18-477E). MacConkey agar was used for cultivation of tested bacteria which were grown aerobically at 37 °C.

#### 2.9.2 Estimation of minimum inhibitory concentration (MIC)

The broth dilution method was used for the determination of MIC according to Clinical and Laboratory Standards Institute guidelines and the protocol mentioned in a previous study with slight modifications.^27^^,^^28^ Briefly, different dilutions were prepared ranging from 400 to 750 μg/mL. A volume of 10 μL of the tested bacteria was inoculated to each concentration of the plant sample. As sterility regulation, Mueller-Hinton broth (MHB, Scharlau) alone was tested while untreated bacteria inoculated on MHB alone were screened as growth control with DMSO (1%). The lowest antimicrobial agent concentration, which inhibited visible growth, was described as MIC. The Minimal Bactericidal Concentration (MBC) was determined by sub-culturing 10 μL from the broth dilution onto Mueller-Hinton agar, ensuring the complete eradication of 100% of the microbes. To ensure accuracy, all assays were conducted in duplicate.

### 2.10 Statistics

The results were provided as the mean value ± SEM, and the number of repeated experiments is denoted by “n". The EC_50_ values (the median effective concentrations) were evaluated together with their corresponding 95% confidence intervals (CI).^29^ Statistical analysis was performed by choosing column statistics from GraphPad Prism software (version 4) with Student's *t*-test or two-way ANOVA, followed by Bonferroni's post-test, to compare concentration-response curves (CRCs) with their respective control group.^20^ In the laxative activity, a comparison was made among groups using one-way ANOVA followed by Dunnett's test. The antidiarrheal effect was assessed statistically by comparing all groups to the control group using the Chi-square (χ^2^) test. A *P* < 0.05 was considered statistically significant. Regression analysis of CRCs was conducted using GraphPad Prism software (version 4).

## 3 Results

### 3.1 Crude extract yield (%)

*M. salicifolia* hydroalcoholic root extract resulted in the final yield of 26.97% (v/w), labeled as Ms.Cr.

### 3.2 GC-MS analysis

**Fig. 1:**
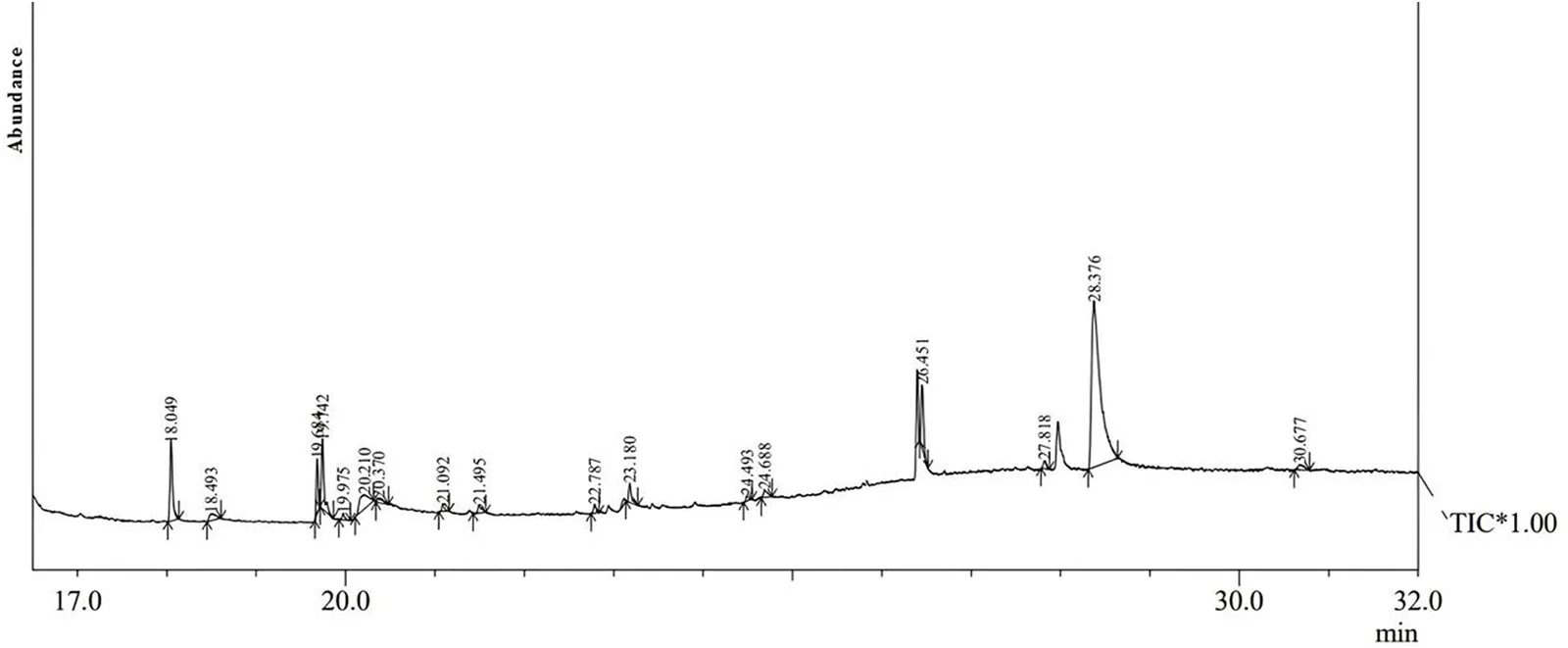
GC-MS chromatogram of crude extract of *M. salicifolia* (Ms.Cr).

The GC-MS analysis showed the presence of 16 different components in Ms.Cr as shown in Table 1 and Fig. 1. The major components found in the extract were methyl hexadecanoate (19.21%), 6-octadecenoic acid, methyl ester (16.23%), cyclohepta [c]furan-4-one, 8-ethoxy-6-(4-hydroxy-3-methoxyphenyl)-1,3-dimethyl (13.85%), cyclododecylmethanol (12.67%), methyl (9Z,12Z)-octadeca-9,12-dienoate (9.91%), methyl 8-(2-octylcyclopropyl) octanoate (4.67%), undecanoic acid (4.01%), and allohimachalol (3.19%). Whereas octadecanoic acid, 2-hydroxy-1-(hydroxymethyl) ethyl ester (2.75%), 13-docosenoic acid (2.52%), chloromethyl 2-chlorododecanoate (2.01%), β-sitosterol monomethyl ether (1.83%) and 1H indole-3-acetic acid (1.38%), etc. were found as minor components of the extracts (Table 1).

**Table 1:** GC-MS analysis of crude extract of *M. salicifolia* (Ms.Cr).

S. No.	Compound Name	RT (Min)	% Area	Chemical formula
1	Methyl hexadecanoate	18.049	19.21404	C_17_H_34_O_2_
2	Undecanoic acid	18.493	4.01526	C_11_H_22_O_2_
3	Methyl (9Z,12Z)-octadeca-9,12-dienoate	19.684	9.91336	C_19_H_34_O_2_
4	6-Octadecenoic acid, methyl ester	19.742	16.23675	C_19_H_36_O_2_
5	Heptadecanoic acid, methyl ester	19.975	1.46630	C_18_H_36_O_2_
6	Cyclododecylmethanol	20.21	12.67924	C_13_H_26_O
7	13-docosenoic acid	20.37	2.52808	C_22_H_42_O_2_
8	(2S)-2-[(2R)-3,4-dihydroxy-5-oxo-2,5-dihydrofuran-2-yl]-2-hydroxyethyl hexadecanoate (L-ascorbic acid, 6-hexadecanoate)	21.092	1.69124	C_22_H_38_O_7_
9	Glycidyl palmitate	21.495	2.54113	C_19_H_36_O_3_
10	Chloromethyl 2-chlorododecanoate	22.787	2.01648	C_13_H_24_Cl_2_O_2_
11	Methyl 8-(2-octylcyclopropyl) octanoate	23.18	4.67518	C_19_H_36_O_2_
12	1H indole-3-acetic acid	24.493	1.38747	C_10_H_9_NO_2_
13	Octadecanoic acid, 2-hydroxy-1-(hydroxymethyl) ethyl ester	24.688	2.75043	C_21_H_42_O_4_
14	Cyclohepta [c]furan-4-one, 8-ethoxy-6-(4-hydroxy-3-methoxyphenyl)-1,3-dimethyl	26.451	13.85067	C_20_H_20_O_5_
15	(3β)-3-methoxystigmast-5-ene (β-Sitosterol monomethyl ether)	27.818	1.83701	C_30_H_52_O
16	(4aR,5R,9aR)-1,1,4a,8-tetramethyl-2,3,4,4a,5,6,7,9a-octahydro-1H-benzoannulen-5-ol (Allohimachalol)	30.677	3.19738	C_15_H_26_O

### 3.3 FTIR studies

An FTIR spectrum was recorded to identify the functional groups and chemical bonds present in the extract, which can be used in comparison in future studies and industrial applications. The major FTIR peaks of extract (Fig. S1) were assigned corresponding to functional groups, -O-H- str of carboxylic acid (3305 cm^−1^), -C-C- str of aromatics (1450 cm^−1^), -C-O- str (1344 cm^−1^) of alcohol, carboxylic acid, esters, ethers and anhydride aromatics, -C-N- str (1204 cm^−1^) of aliphatic amines, -N-H- str (728, 827, and 1696 cm^−1^) of primary and secondary amines, amides and carboxylic acid.

### 3.4 Laxative effects

Ms.Cr treatment produced 61.34 ± 2.87% and 84.01 ± 4.05% wet feces in mice at doses of 50 and 100 mg/kg, respectively. Administration of CCh (1 mg/kg) in the positive control group produced 85.71 ± 3.08% wet feces, while no wet feces were observed in the saline-treated group. In the atropinized mice, the Ms.Cr (50 & 100 mg/kg) effects on the count of wet feces reduced to 37.83 ± 4.72% and 44.04 ± 5.02%, respectively (Table 2).

**Table 2:** Laxative effect of the crude extract of *M. salicifolia* (Ms.Cr) in the absence and in the presence of preatropinized mice.

Group No.	Treatment	Dose (mg/kg)	Defecation/group	Number of wet feces/group	% of wet feces
1	Saline (PO, mL/kg)	10	3.16 ± 0.30	0	0
2	Carbachol (IP)	1	11.83 ± 0.30**∗∗**	10.16 ± 0.54**∗∗∗**	85.71 ± 3.08
3	Ms.Cr (PO)	50	8.61 ± 0.80**∗∗**	5.33 ± 0.61**∗**	61.34 ± 2.87
4		100	11.66 ± 1.20**∗∗**	9.66 ± 0.76**∗∗**	84.01 ± 4.05
5	Carbachol (IP) + Atropine (IP)	1 + 10	4.16 ± 0.47**^##^**	0.83 ± 0.30**^###^**	20.00 ± 7.93
6	Ms.Cr (PO) + Atropine (IP)	50 + 10	5.83 ± 1.01**^†^**	2.00 ± 0.25**^†^^†^**	37.83 ± 4.72
7		100 + 10	6.32 ± 0.60**^@@^**	2.50 ± 0.34**^@@@^**	44.04 ± 5.02

Data are expressed as mean ± SEM, n = 6. ∗*P* < 0.05, ∗∗*P* < 0.01 and ∗∗∗*P* < 0.001 show a comparison of group no 2, 3 and 4 vs. group no 1 (One-way ANOVA followed by Dunnett's test), ##*P* < 0.01 and ###*P* < 0.001 group no 5 vs. group no 2. †*P* < 0.05 and ††*P* < 0.01 group no 6 vs. group 3 and @@*P* < 0.01 and @@@*P* < 0.001 group no 7 vs. group no 4 (unpaired *t-*test).

### 3.5 Antidiarrheal effects

Table 3 shows the dose-dependent antidiarrheal effect of Ms.Cr (200 and 400 mg/kg) against castor oil-induced diarrhea in mice, while the saline group did not show any protection. The mice administered with lower dose (200 mg/kg) exhibited 40% while 80% diarrheal protection was seen with higher dose (400 mg/kg) of Ms.Cr. The standard antidiarrheal drug (loperamide) did not show any diarrheal spots and thus resulted in 100% protection (Table 3).

**Table 3:** Antidiarrheal effect of the crude extract of *M. salicifolia* (Ms.Cr) on castor oil-induced diarrhea in mice.

Treatment	No. of mice (with diarrhea)	% Protection
Saline (10 mL/kg, PO) + Castor oil (10 mL/kg)	5/5	0
Ms.Cr (200 mg/kg) + Castor oil (10 mL/kg)	3∗/5	40
Ms.Cr (400 mg/kg) + Castor oil (10 mL/kg)	1∗/5	80
Loperamide (10 mg/kg) + Castor oil (10 mL/kg)	0∗∗/5	100

∗*P* < 0.05 and ∗∗*P* < 0.01 vs. Saline + Castor oil treated group (χ^2^-test).

### 3.6 Gut stimulatory effects

**Fig. 2:**
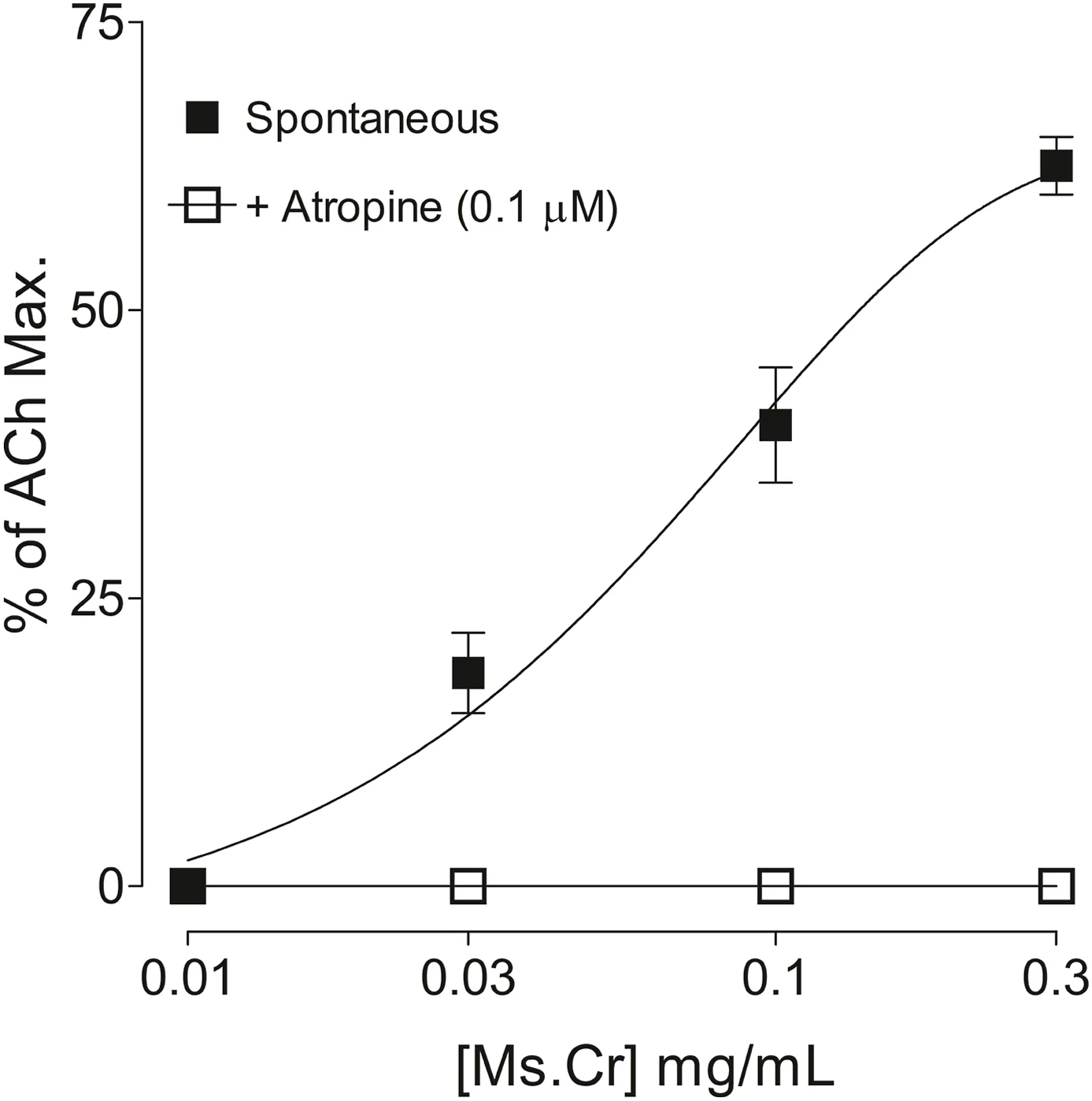
Dose-response curves of the spasmogenic effect of the crude extract of *M. salicifolia* (Ms.Cr) in the absence and presence of atropine (0.1 μM), in isolated rabbit jejunum preparations. Data are expressed as mean ± SEM (n = 6) and showed as the percentage of ACh maximum response.

The crude extract of Ms.Cr caused a concentration-mediated excitatory response on the spontaneous contractions of rabbit jejunum at concentrations of 0.03, 0.1, and 0.3 mg/mL with maximum contractile peak as 62.44 ± 2.50% (mean ± SEM; n = 6) of ACh maximum contractile effect. Pretreatment of the tissue with atropine (0.1 μM) completely abolished the contractile effect of Ms.Cr (Fig. 2).

### 3.7 Gut inhibitory effects

**Fig. 3:**
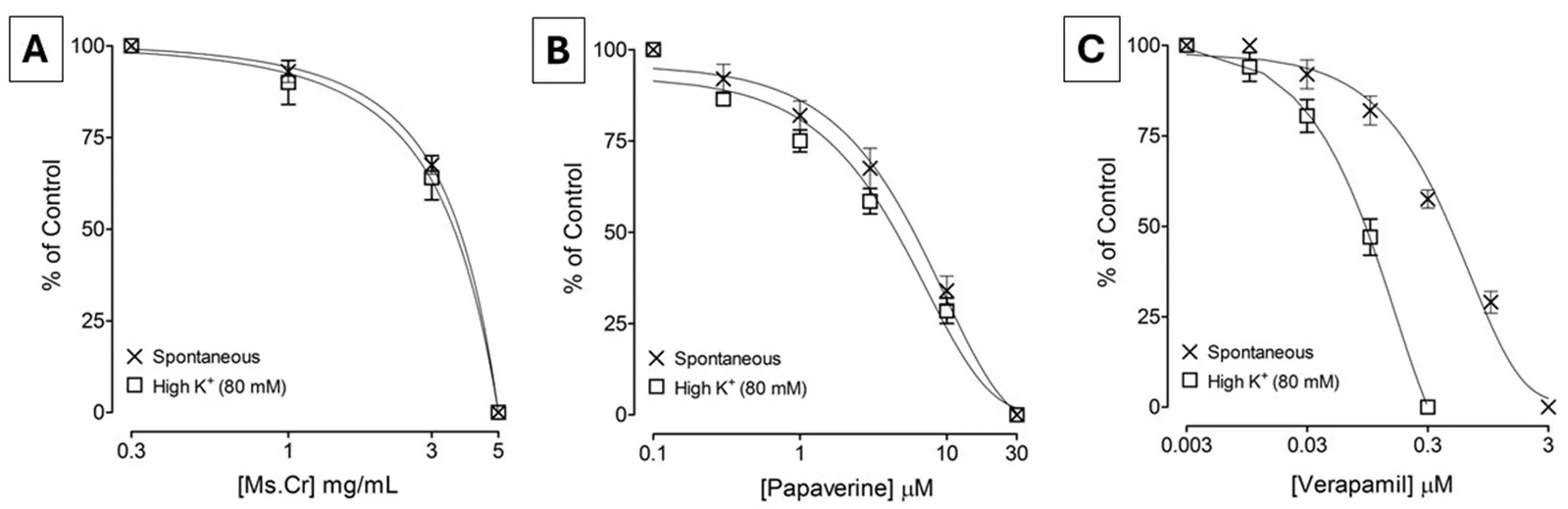
Concentration-response curves of the **(A)** crude extract of *M. salicifolia* (Ms.Cr), **(B)** papaverine, and **(C)** verapamil, for the inhibitory effect on spontaneous and high K^+^-induced contractions in isolated rabbit jejunum preparations. Data are presented as mean ± SEM, n = 6.

Ms.Cr caused dose-dependent (1.0-5.0 mg/mL) inhibition against spontaneously contracting and high K^+^-induced contractions in jejunal strips with resultant EC_50_ values of 3.48 mg/mL (3.28-3.94, 95% CI, n = 6) and 3.26 mg/mL (3.04-3.76, 95% CI, n = 6), respectively (Fig. 3A). Similarly, papaverine also exhibited comparable relaxant effects on both spontaneous and high K^+^ (80 mM)-induced contractions, with EC_50_ values of 7.44 μM (7.04-7.92, 95% CI, n = 6) and 7.22 μM (6.84-7.62, 95% CI, n = 6), respectively (Fig. 3B). In contrast, verapamil demonstrated significantly greater potency against high K^+^ compared to its inhibitory effect on spontaneous contractions, with resultant EC_50_ values of 0.22 μM (0.18-0.28, 95% CI, n = 6) and 2.42 μM (2.18-2.68, 95% CI, n = 6), respectively (Fig. 3C).

**Fig. 4:**
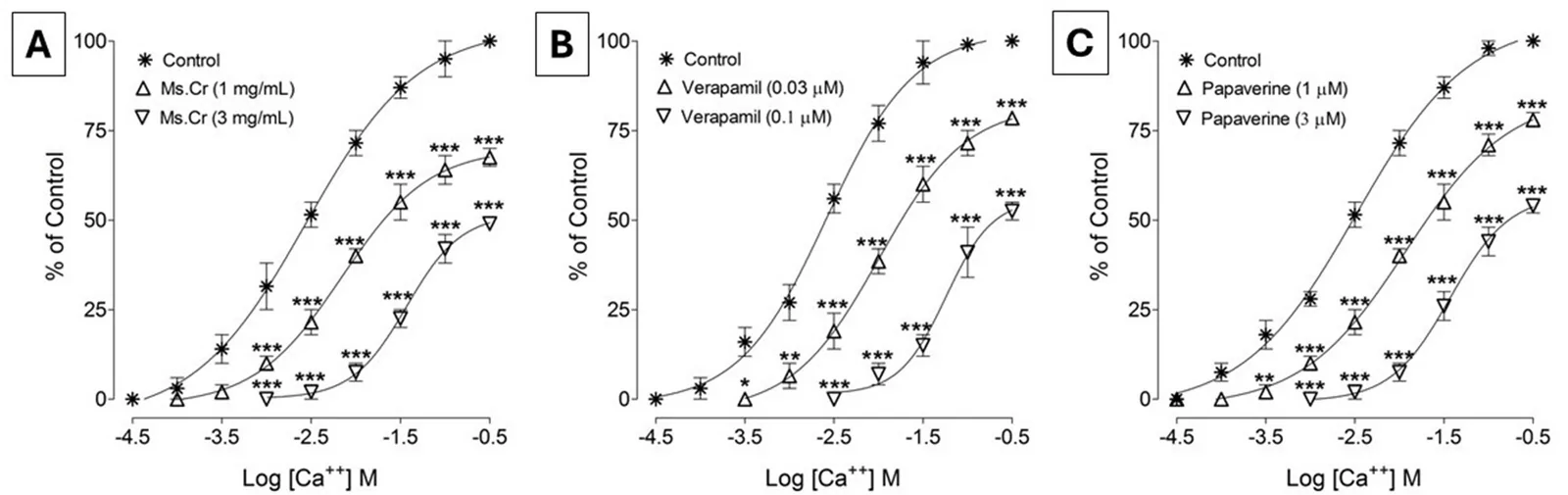
Concentration-response curves of Ca^++^ constructed in the absence and presence of the increasing concentrations of the **(A)** crude extract of *M. salicifolia* (Ms.Cr), **(B)** verapamil, and **(C)** papaverine in isolated rabbit jejunum preparations. Data are expressed as mean ± SEM, n = 6. ∗∗*P* < 0.01, and ∗∗∗*P* < 0.001 compared to respective concentration values in Ca^++^ control (untreated) curves, two-way ANOVA followed by Bonferroni post-test.

When further tested, jejunal tissues pre-incubated with Ms.Cr (1.0 and 3.0 mg/mL) shifted the Ca^++^ CRCs towards right with suppression of the maximum response and thus confirmed the Ca^++^ inhibitory effect of Ms.Cr (Fig. 4A). Verapamil, a standard Ca^++^ inhibitor, also suppressed and shifted Ca^++^ CRC towards right in a non-specific manner (Fig. 4B). Papaverine (1 and 3 μM) showed similar displacement of Ca^++^ curves to verapamil (Fig. 4C).

**Fig. 5:**
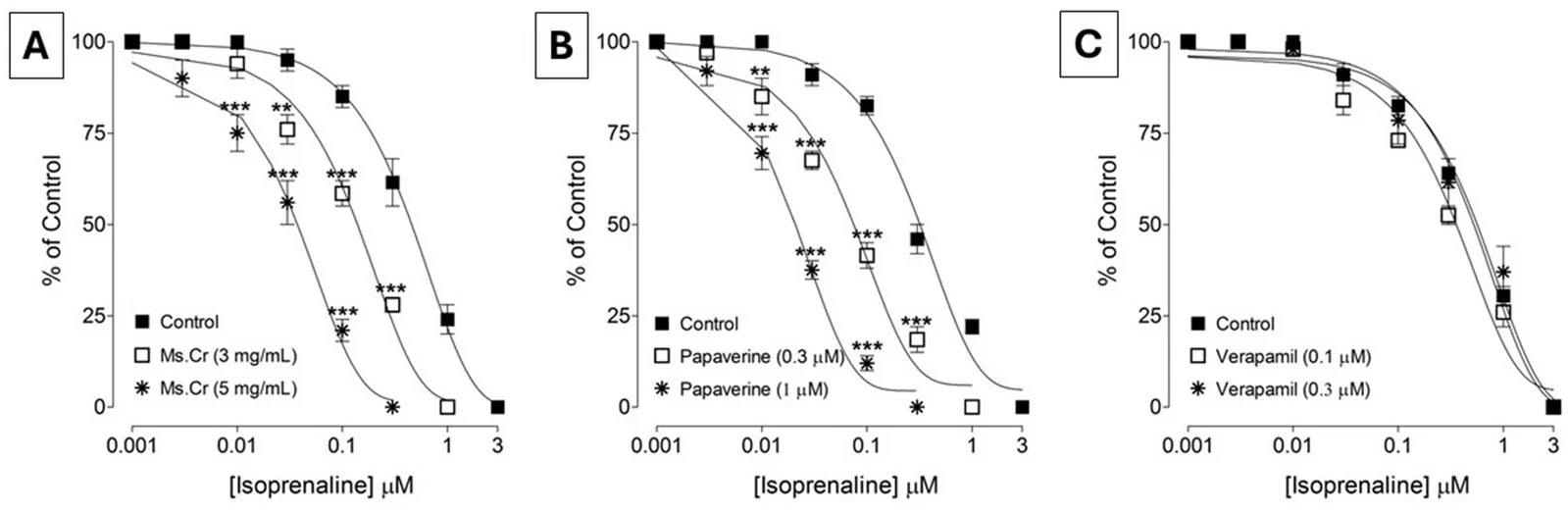
Inhibitory concentration-response curves of isoprenaline against carbachol (CCh)-induced contractions in the absence and presence of different concentrations of **(A)** crude extract of *M. salicifolia* (Ms.Cr), **(B)** papaverine and **(C)** verapamil, in isolated rabbit jejunum preparations. Data are expressed as mean ± SEM, n = 6. ∗∗*P* < 0.01, and ∗∗∗*P* < 0.001 compared to respective concentration values in isoprenaline control (untreated) curves, two-way ANOVA followed by Bonferroni post-test.

To confirm PDE-inhibitory activity indirectly, pretreatment of tissues with Ms.Cr (3.0 and 5.0 mg/mL) shifted the isoprenaline-induced inhibitory CRCs constructed against CCh towards left, indicating a potentiating effect (Fig. 5A). Similar leftward potentiation of the isoprenaline curves was observed with papaverine (0.3 and 1 μM), as shown in Fig. 5B. In contrast, verapamil did not show potentiating effect (Fig. 5C).

### 3.8 Antibiotic susceptibility pattern of ESβL-*E. coli*

In the current study, ESβL- *E. coli* as a clinical pathogen was used and its antibiotic susceptibility pattern was authenticated in King Khalid Hospital using a Phoenix 100/BD company machine. The results were evaluated following the guidelines set by the Clinical and Laboratory Standards Institute (CLSI). ESβL- *E. coli* was resistant to Cefotaxime, Amoxicillin/clavulanate, Ampicillin, Cefuroxime, Cefepime, Ceftazidime and Aztreonam.

The MIC and MBC of Ms.Cr are shown in Table S1. There was no difference in sensitivity observed among the standard tested bacteria (MIC 550 μg/mL). While ESβL- *E. coli*, was more resistant (MIC 675 μg/mL) than other reference tested bacteria.

## 4 Discussion

The hydroalcoholic root extract of *M. salicifolia* was evaluated for potential gut modulatory effects, taking into account its therapeutic application in various health-related problems. The *in vivo* studies conducted in mice showed that lower doses of Ms.Cr pretreatment caused an increase in number of wet stool, thereby exhibiting laxative effect similar to the effect seen with carbachol, an agonist at muscarinic subtype of cholinergic receptor and accelerator of intestinal content.^30^ These intestinal stimulatory effects of Ms.Cr were found partially sensitive to atropine, a non-specific muscarinic receptor blocker,^21^ thus suggesting the presence of ACh-like components in Ms.Cr in addition to some unexplored mechanisms. ACh, a neurotransmitter in the parasympathetic nervous system, is known for increasing gut motility through activation of muscarinic receptors,^30^ therefore, Ms.Cr therapeutic role as a digestive aid and in constipation is demonstrated by the presence of ACh-like constituents. The co-existed unexplored gut stimulatory mechanisms present in Ms.Cr might include certain prostaglandins,^31^ platelet-activating factor,^32^ nitric oxide-releasing compounds,^33^ dopaminergic antagonist,^34^ cholecystokinin,^35^ and tachykinins,^36^ as these agents are known for laxative effects. The cholinomimetic-like mechanism involved in the gut stimulatory effect of Ms.Cr was further supported by the *ex vivo* experiments conducted in isolated rabbit jejunum preparations.^37^ Interestingly, Ms.Cr, at lower concentrations, showed atropine-sensitive stimulant effect on the spontaneously contracting rabbit jejunum, similar to ACh, thus authenticating the involvement of muscarinic-like constituents in Ms.Cr.

The preliminary identified phytoconstituents of *M. salicifolia* might be linked to the observed gut stimulatory effects as both the alkaloids and saponins have been reported for gut stimulation,^38^^,^^39^ though additional detailed assays are recommended to know the precise role of these active constituents. *M. salicifolia* has also been reported in traditional system for the treatment of diarrhea and hyperactive gut spasms,^4^ therefore, experiments were extended to possibly evaluate the plant extract in castor oil-induced diarrhea model (*in vivo*) while isolated jejunal preparations were used in *ex vivo* assays (as stated earlier) for the exploration of antispasmodic mechanisms.^40^ The castor oil-induced diarrhea is a well-known model in laboratory animals to explore the antidiarrheal potential of unknown sample as it induces copious diarrhea after hydrolyzing to ricinoleic acid in the gut.^41^ Pretreated mice with Ms.Cr at higher doses of 200 and 400 mg/kg showed dose-dependent diarrheal protection similar to loperamide, a common antidiarrheal drug.^42^ After observing the antidiarrheal effect, the next objective was to evaluate the possible mechanism by testing the higher concentrations of Ms.Cr against the spontaneous contractions of jejunum.^43^ Interestingly, Ms.Cr exhibited complete inhibition of spontaneous contractions at concentrations higher to the earlier tested concentrations where spasmogenic effect was observed. Based on earlier studies, spasmolytic agents inhibit gut peristaltic movements by blocking either the Ca^++^ channels,^42^ and/or PDE inhibition,^44^^,^^45^ hence Ms.Cr was tested against high K^+^-induced contractions in jejunal tissues where it caused complete relaxation.^46^ The critical examination of the pattern of inhibitory CRCs of Ms.Cr against high K^+^ and spontaneous contractions reveals no statistical difference (*P* > 0.05) in their potency, similar to papaverine, a dual inhibitor of Ca^++^ channels and PDE.^23^ On the other hand, substances such as verapamil that selectively show higher potency against high K^+^ compared to its effect against spontaneous contractions are considered CCBs.^47^^,^^48^ Any substance that reverses high K^+^ (> 30 mM)-induced contractions is considered as CCBs,^49^ and to further confirm this CCB-like activity present in Ms.Cr, jejunal tissues were made Ca^++^ by stabilizing in Ca^++^ free buffer solution. The contractile CRCs of Ca^++^ were made in the absence and in the presence of the pre-incubated jejunal tissues with increasing doses of Ms.Cr that caused deflection in the Ca^++^-CRCs towards right with suppression of the maximal response, similar to verapamil, thus confirming CCB-like action. The presence of additional PDE inhibitory-like components in Ms.Cr was indirectly confirmed by isoprenaline inhibitory CRCs constructed against CCh as PDE inhibition are known to cause CCh relaxation by increasing the tissue cAMP levels. Tissue PDE is considered to convert active cAMP to inactive AMP by chemical reaction and thus substances that inhibit PDE will consequently cause an increase in the levels of active cAMP in smooth muscles and results in antispasmodic effect.^50^ The leftward shift observed in the inhibitory CRCs of isoprenaline towards lower concentration in the isolated tissues is an indication of the potentiation effect and thus indirectly supports the presence of PDE inhibitory-like components in Ms.Cr, similar to papaverine, a known PDE inhibitor.^51^^,^^52^

For the dose selection, we followed the earlier report by Ndanyi et al.^53^ where *M. salicifolia* root extract was found safe up to the maximum tested dose of 2000 mg/kg in rats and mice.^38^ While evaluating plant extracts, it is interesting that some plants possess co-existence of gut stimulant and inhibitory components by exhibiting laxative effect usually at lower doses (50 and 100 mg/kg), followed by antidiarrheal activities at higher doses (100 and 200 mg/kg), such as cardamom,^54^ *Lavandula* stoehas,^55^ psyllium husk,^56^ *Phyllanthus emblica*,^57^ ginger,^37^ and *Lepidium sativum*.^20^^,^^58^ The gut stimulatory efficacy of Ms.Cr observed in current study when compared with cardamom, lavandula and psyllium husk was found to be higher (62.44%) than lavandula (57.1%),^55^ whereas lower than psyllium husk (71.2%),^56^ and cardamom extract (70.61%),^54^ whereas the relaxant potency of Ms.Cr is found comparable to cardamom extract while both lavandula and psyllium husk exhibited higher potencies. It appears that the co-existence of gut modulatory constituents in Ms.Cr is probably meant by nature to offset the gut stimulant effect beyond which it could be harmful by causing abdominal cramps as seen with the pure chemical drugs used in constipation.^23^ This is in line with the general perception that natural products possess “synergistic and/or side effects neutralizing components".^2^

Common gut infectious agents including Gram-negative bacteria have an outer membrane as part of their cell wall structure, which is composed of lipopolysaccharides that become a hydrophilic surface and thus restrain the permeability of plant extracts, detergents, lipophilic compounds, and/or antibiotics.^59^ Furthermore, there is a gradual increase in the resistance rates of these pathogens, including extended-spectrum β-lactamase (ESβL) producing pathogens such as *E. coli*. This poses a significant threat to public health and has exacerbated the management of bacterial infections. The antibacterial effectiveness of *M. salicifolia* in this study showed that ESβL producing *E. coli* was more resistant than the standard strains of *E. coli, Sh. sonnei* and *S. typhimurium*. We speculate that the fatty acid esters identified by the GC-MS are associated with the recorded bioactivity of Ms.Cr. Some previous studies described the antimicrobial and antimycobacterial activities of fatty acid esters.^60^, ^61^, ^62^, ^63^ Moreover, methyl hexadecanoate, the major compound found in Ms.Cr, has been reported as anti-inflammatory and antifibrotic,^64^ and these effects can be further support in the symptoms of gut bacterial infections such as inflammation and fever associated with diarrhea. Additionally, β-Sitosterol identified in the Ms.Cr may also contribute in the antibacterial effect of the plant extract as β-Sitosterol is known for its antimicrobial effect.^65^ In the current study, we did not explore the possible mechanisms for the exerted antibacterial effect of Ms.Cr, however by decline in the internal pH and membrane potential hyperpolarization in the tested strains of bacterial cells might be the potential target of Ms.Cr.^66^ Additionally, fatty acids, both saturated and unsaturated are known for their antibacterial activity,^67^ and might play role in the antibacterial activity of Ms.Cr. Saturated fatty acids, such as palmitic acid, is believed to act by inducing ROS, thereby increasing cellular toxicity and impairment of bacterial cell membrane function,^68^ while unsaturated fatty acids, linoleic acid has shown antibacterial activity against Gram-positive bacteria by inhibiting cell wall biosynthesis and loss of energy production in *S. aureus* by altering the gene expression of the glycolytic and fermentative metabolic pathways.^69^^,^^70^ Further, β-Sitosterol has been found to inhibit the biosynthesis of peptidoglycan and prevent bacterial cell wall formation by inhibiting the key enzymes MurA and SrtA.^71^ The diarylheptanoids reported from *M. salicifolia* have shown antibacterial activity possibly by exerting damage to the bacterial membrane, inhibition of bacterial biofilm production and virulence factors.^72^

In the GC-MS analysis of the plant extract, a total of 16 compounds were found including different fatty acids and their ester derivatives. Among these, allohimachalol identified in *M. salicifolia* might partly be responsible for the observed antispasmodic effect of Ms.Cr as another similar compound himachalol, a stereoisomers of allohimachalol has been reported to possess antispasmodic and smooth muscle relaxant effects in guinea pig ileum, rabbit jejunum, rat uterus, and guinea pig seminal vesicle mediated possibly by antimuscarinic, antihistaminic, and activity similar to that of papaverine.^73^ Interestingly, both these sesquiterpenes share the same molecular formula and fundamental structure, but their atoms are arranged differently in space. Another compound, linoleic acid has been reported to ameliorate high-fat diet-induced gut barrier damage by modulating gut metabolism.^74^ Moreover, β-Sitosterol identified in Ms.Cr might also support the gut modulatory effect of this plant as β-Sitosterol has been reported to possess antispasmodic effect in isolated rabbit jejunum mediated by Ca^++^ channel inhibition,^75^ in addition to its anti-inflammatory, antioxidant,^76^ anticancer and colitis-protective,^77^ cardioprotective, antidiabetic, hepatoprotective,^78^ and antimicrobial.^65^

Despite the strong gut modulatory effects presented, the current study has some limitations which will be addressed in our future studies on this plant. The major limitation is lack of quantification of the key bioactive compounds identified in the extract of this plant as it is considered for quality control and can further support the scientific validity and reproducibility of the findings. Moreover, the biological evaluation of the identified compounds has not been conducted which otherwise would have provided more support to our current findings. Secondly, the antimicrobial activity was conducted in *in vitro* that need further detailed *in vivo* investigation to prove the possible protective effect of Ms.Cr in an infectious animal model. Thirdly, this study would have more clinical impact if human data related to pharmacokinetic profile of this plant been investigated in addressing gut modulatory disorders. Further, in our *in vivo* experiments, the dose selection was based on the earlier reported toxicity study conducted in rat, however, it is recommended to conduct safety studies on more than one species before a potential compound can be developed for human use as multiple studies show specie and tissue-specificity present in different plant extracts.^20^^,^^46^^,^^79^

The pharmacological effects of Ms.Cr are supported by functional assays demonstrating muscarinic-mediated contraction, Ca^++^ channel blockade, and PDE-related relaxation, providing direct evidence for its biphasic activity. GC-MS–identified constituents (e.g., fatty acids, β-sitosterol) have reported modulatory effects and may partly contribute, although this linkage remains literature-based rather than experimentally confirmed.

In conclusion, the gut modulatory effects of Ms.Cr can be explained by a biphasic, dose-dependent mechanism. At low concentrations, Ms.Cr enhances gut motility via muscarinic receptor activation, leading to smooth muscle contraction. At higher concentrations, it induces relaxation through Ca^++^ channel blockade and PDE inhibition, reducing intracellular Ca^++^ and increasing cAMP. This bidirectional action suggests multiple active constituents acting on different targets, enabling a shift from spasmogenic to spasmolytic effects and supporting its traditional use in regulating gut motility. However, we recommend further sophisticated experiments to precisely know the involvement of additional unexplored mechanisms.

## Authors contribution

NUR: conceptualization, data curation, formal analysis, funding acquisition, investigation, project administration, writing original draft, review and editing, MNA: Analysis, writing, review and editing, AK: conceptualization, investigation, writing, review and editing, ART, HMD and KYA: investigation, writing, review and editing, WA and MUR: analysis, writing, review and editing.

## Funding

The authors extend their appreciation to 10.13039/100009392Prince Sattam bin Abdulaziz University for funding this research work through the project number (PSAU/2024/03/29633).

## Declaration of competing interest

The authors declare that they have no known competing financial interests or personal relationships that could have appeared to influence the work reported in this paper.
