# Supplementary material for: Pharmacological basis for the traditional use of Myrica salicifolia root extract in gut motility disorders via cholinergic and calcium/PDE modulation
Source: J Tradit Complement Med. 2026 May 28;16(5):547–55. doi: 10.1016/j.jtcme.2026.05.001 (PMC13541586; doi:10.1016/j.jtcme.2026.05.001)
Supplement: Supplementary file 1 [file mmc1.docx]

**Fig. S1.** FTIR spectra of crude extract of *M. salicifolia* (Ms.Cr).

Table S1. MIC and MBC of the crude extract of *M. salicifolia* (Ms.Cr) vs. Tested Bacteria.

| **Bacteria** | **MIC** | **MBC** |
| --- | --- | --- |
| *E. coli* (ATCC 11209) | 550 µg/mL | 575 µg/mL |
| *Sh.* sonnei (ATCC 11060) | 550 µg/mL | 575 µg/mL |
| S*.* typhimurium (ATCC 14025) | 550 µg/mL | 575 µg/mL |
| *ESBL *E. coli* | 675 µg/mL | 700 µg/mL |

* Clinical bacteria
